# International Dental Federation and International Commission of Education: Meeting Held in Stockholm, Sweden, August, 1902

**Published:** 1903-05

**Authors:** 


### INTERNATIONAL DENTAL FEDERATION AND INTERNATIONAL COMMISSION OF EDUCATION: MEETING HELD IN STOCKHOLM, SWEDEN, AUGUST, 1902.

(Continued from page 234.)

*The President.*—The general discussion this afternoon embraces three topics prepared last year for our consideration. The first is, "What are the preliminary studies to be required of students before beginning their professional education?" I will ask Professor Hesse, of Leipzig, to open the discussion.

*Professor Hesse* (speaking in German).—All I have to say on this question is printed in the first of the three fasciculi issued by the Federation, and I expect that every one will have read them, and anything I might say would be simply repeating what is already printed; therefore I prefer to say nothing now.

The following is an extract from Professor Hesse's report:

### REPORT TO THE INTERNATIONAL COMMISSION OF EDUCATION, BY PROFESSOR DR. HESSE, OF LEIPZIG, GERMANY.

Following the decision of the International Commission of Education, I have the honor of submitting the following answers to the questions proposed by the Commission:

*First:* "What should be the preliminary requirements for admission into dental schools?"

*Answer:* It should be similar to that required of medical students.

*Second:* "What should be the length of time devoted to the study of dentistry, and the arrangement of the curriculum?"

*Answer:* The duration of the course should be of three years at least. The curriculum should comprise—(*a*) Natural science,—namely, physics and inorganic chemistry; (*b*) Medical sciences,—viz., anatomy, physiology, general pathology, bacteriology, embryology, general surgery, and materia medica; (*c*) The science and art of dentistry. In these studies a prominent place should be given to practical dental work. During all the time devoted to the various divisions of the course the dental student should be required to devote at least three hours daily to practical operative and technical studies. This course would have for the student the same value as the attendance at hospitals has for the medical student.

*Third:* "What part of the subjects that are taught in medical schools should be pursued by the dental student?"

*Answer:* The dental student should follow a general course in physics and chemistry, and should also follow a course in general anatomy, general pathology, embryology, and bacteriology. We recommend that a special course in general anatomy, physiology, general pathology, and materia medica should be given to dental students, as we deem that these topics embrace many features which are of no value to the dentist, and which cannot be acquired in the lapse of time at the disposal of the dental student.

Dr. Queudot, who spoke in French, was of the opinion that it is impossible to have a good dentist unless he is an educated man. The preliminary instruction is especially important, and in the professional education could be included such subjects as bacteriology and others that are included in a medical curriculum but are not really medical. Only the best is good enough for dentists. After that the student may take up his work in the dental school, and take it up with a light heart, because he has been well trained and has a stiff leg and a strong arm. What was difficult becomes easy because of the good training he has had before.

*Dr. B. Holly Smith.*—My own impression is that the work we have to do this afternoon is the discussion of Dr. Brophy's address and Dr. Barrett's paper, and I will come directly to that. The education of the dentist must be separated from that of the medical man. I ask you to consent to the discussion proceeding on those lines, because however broadly the foundation of medical education is laid, however widely and definitely the plans are made for the education of the student who attempts to practise dentistry, medical instruction is lost time. Start your student to study anatomy, physiology, bacteriology, therapeutics, and materia medica in the dental school, but do not send him to men who are teaching general anatomy, general pathology, and general physiology. Put him absolutely under the control of competent, capable, cultured people who know his specialty, and who will train him for it. I say that because life is short. We begin too late to do our life's work. Thirty-one years! You cannot start practising so late as that. How much of life has gone in those thirty-one years! If you propose to have a medical education, a dental education, and a degree from a literary institution, you are thirty-one years old when you start to practise dentistry, and we cannot have that. It is extremely embarrassing to me that I cannot understand the question.

*Dr. Aguilar.*—You have understood it.

*Dr. Harlan.*—Perfectly.

*Dr. Cunningham.*—Not at all: I protest! Did you say, Dr. Queudot, that a dentist must have a medical education?

*Dr. Queudot.*—No.

*Dr. B. Holly Smith.*—I want to say with regard to the very paper of my dear friend and beloved *confrère,* Dr. Brophy, that I admire many of the elucidations of what we consider in America some intricate problems in education. I admire it because as a teacher of long experience he has determined a course. In the city of Stockholm I have admired as much as anything else the beauty of the water, the lovely architecture, and the lovely display of nature's gifts, but I have admired one thing above all, the quickness and promptness of the driver, the man who knows the way, the man who goes quickly, who steers clear of disaster. My dear friend Dr. Brophy has opened the way for us with regard to education, and that is the way we must follow. A university can never teach dentistry and medicine together. If we establish a course in dentistry and have professors of medicine, professors of physiology, and professors of chemistry, and the student studies dental chemistry, dental physiology, and dental anatomy from his own stand-point, then it is possible, but it is not possible when you say that a dental student may slink in through the door and may take what he can get from a medical man by the side of the medical student, and then when the time comes for examination he is told, "Oh, you are only a dental student!" We cannot have that! I do not say that a man, because he is a medical man, could not prepare himself to teach these students, but you cannot have medicine and dentistry taught together. I am opposed to it. Dr. Barrett made a remark that Dr. Harris had endeavored to establish a dental course, and said that that was an incident which was unimportant.

*Dr. Barrett.*—I did not say such a thing; I said that it was merely an incident that had occurred in America, but it might have occurred anywhere else. It had nothing to do with the merits of the case that it occurred in America first.

*Dr. B. Holly Smith.*—I stand here as the representative of the Baltimore College of Dental Surgery, the first and oldest college in the world, and I say that dentistry must feel grateful that Chapin A. Harris and Horace Hayden established a dental school. Why do I say so? I feel the absolute importance to be attached to the beginning of dentistry, and I think there is no excuse whatever for passing that by as an unimportant question. Dentistry began in Baltimore, and dentistry will continue throughout the world. There never will be a time when dentistry will lose its identity as an individual and distinct calling.

*The President.*—It is desirable, if we can, to limit our discussion as far as possible to the three general questions. I do not think it is expedient to pursue further the remarks on the papers that have been presented. We ought to dispose this afternoon of the discussion of the papers that have been presented, and when we meet together again we can then take up the three general questions.

*Dr. George Cunningham.*—While I do not desire to make any speech at all or go into the merits of the question, I want to make you acquainted with the fact that, owing to work which I have to do, I cannot be present, and I should like to take this opportunity of telling you that a great reform has taken place in England with regard to preliminary education. At the present moment they are discussing the Education Bill in Parliament, and that bill will make a great deal of difference to the future history of England. There is a great reform which has taken place, and the nation has not yet learned it. The new University of London has decided that Latin will no longer be compulsory on the medical, dental, or other professional student, and you will see from that that a great step has been taken.

*Dr. Barrett.*—I do not want to be misunderstood. That Chapin A. Harris was an American was merely an accident, and has nothing to do with the question. He might have been an Englishman, a Frenchman, a German, or a Spaniard, and it would not have changed matters or made any difference. I said it was merely an incident that he was an American. I am an American, but my sympathies do not stop with America by any means. We have good American schools, but they have good schools elsewhere. In America I think in some respects we are superior, but there are schools in other countries which in some respects are superior to ours. The question is, May, with propriety, dentistry be taught in the medical schools? It depends on the dentistry. With us the mechanical is the dentistry, and cannot be taught in a medical school. In many countries it is medical teaching, and should be taught in a medical school. My point is, Is there not a common ground on which we can meet,—English, French, German, Swedish, American, under the different systems,—some common system, so that when an American shall desire to enter a German school he may receive the credit for that which he has accomplished, and a German in an American school may also receive the credit for his work? I do not think it would be wise to permit a German to practise in America without any restraint, or an American to practise in Germany. There must be the national regulations to be observed, and hence if a German comes to America he should take a term in an American school and become acquainted with our methods. If an American goes to Germany it may be best that he should take a term in a German school. Is there not some middle ground which would be common to all of us on which we could all meet and shake hands,—German, French, English, and American together?

The discussion was continued by Professor Franck and Dr. Guerini in German and French respectively.

*Dr. Aguilar,* Madrid.—I believe that we are here now for the discussion of three points on which I think resolutions should be taken. Therefore I shall move to present certain resolutions.

*The President.*—Let me suggest that inasmuch as the three questions have not yet been considered, these resolutions are not yet appropriate.

*Dr. Aguilar.*—What are we discussing?

*The President.*—The three questions will be discussed.

*Dr. Aguilar.*—We are discussing them now.

*The President.*—No; we are discussing the address of the President and the paper of Dr. Barrett.

*Dr. Aguilar.*—Then I will say nothing.

*Dr. Guilford.*—Will this subject be continued on Monday?

*The President.*—This is a subject to which we might devote some time on Monday. It is absolutely necessary that the three subjects before us should be considered. They were proposed last year and are before the Commision for discussion, for further consideration, and for adoption. These questions may, however, be discussed if the gentlemen will meet promptly at nine o'clock. I would suggest that instead of coming to this Institution we convene in the Grand Hotel where we are stopping, and thereby save much time.

*Dr. Guilford.*—I would suggest that we confine ourselves to the discussion of the questions themselves. We are not here to discuss the beginnings or history of dentistry or anything else.

*The President.*—This afternoon the question was the address, and after that subject has been disposed of comes the consideration of the three questions, and when we come to the consideration of the three questions other topics will not be discussed.

*Professor Hesse.*—I should not like to leave this room without saying one word of thanks to Professor Brophy, not only for presiding so well, but for his excellent paper, and especially for having it printed in German and in French. That is a great advantage, for our greatest barrier of separation is in not understanding each other's language.

*The President.*—I thank Professor Hesse for what he has said, and I would like at the same time to say that the translation of my address is not accurate. Some errors have been made that may slightly change its meaning, and I would therefore like to have those who have read the address in French and German withhold their judgment upon it until they see it printed in an accurate translation. Some words have been omitted here and there. I think it is eminently fitting that whoever reads an address in English should have it printed in two or three languages. It is a simple thing and it helps so much in coming to an understanding of what the author intends to convey.

Adjourned to meet at nine A.M. Monday, August 18, at the Grand Hotel.

### INTERNATIONAL COMMISSION OF EDUCATION.

*Monday, August 18, 1902.*

Dr. Queudot, Paris, presented a report on "Dental Education" on behalf of the École Odontotechnique, of Paris. In substance he said that dental surgery is a branch of medicine, and that during the preliminary education of the future dental candidate nothing should be neglected that could in any way develop the literary, scientific, and artistic sense of the student. Like the physician, the dentist should possess a good basal knowledge of literature and science, as these are important factors in carrying out the duties of his calling.

Dental education, he claimed, comprises two distinct parts,—namely, the scientific group and the technical group of studies. The technical group is the application of the knowledge acquired from the scientific group. The first duty of the dental student should be to acquire a sufficient degree of knowledge of anatomy, histology, pathology, bacteriology, and of all other branches which, strictly speaking, cannot be called medicine, but which are, however, the basis of medicine and of all its specialties. After acquiring the necessary preparatory education referred to, and only then, should the student be permitted to enter upon the study of the dental branches, in our clinics as well as in the several dental schools.

Dr. Vincenzo Guerini, the official delegate of the government of Italy, then presented the report which here follows:

REPORT TO THE INTERNATIONAL COMMISSION OF EDUCATION BY DR. VINCENZO GUERINI, ITALY.

GENTLEMEN,—I wish to bring before your consideration several general ideas regarding the subject of dental education, but before doing so I must refer to a special feature of dental education in the country which I represent, as this is in strict accord with the ideas which I will now discuss. For about twelve years there has been a law in Italy which requires that all dentists must be holders of the degree of doctor of medicine before they can be allowed to practise their profession. This legislative measure presents great inconveniences, as I have on several occasions endeavored to demonstrate. I have pointed out the necessity that odontology should be taught as a separate and autonomous science, and not as a simple branch of general medicine. I found myself in the necessity of discussing this topic with the *médecins-dentistes,* and as soon as this was taken up I found that in order to defeat my adversaries the first thing I had to do was to recognize whatever truth could be found in their arguments, this being in my estimation the only way of clearly demonstrating the fallacy of their conclusions.

In the arguments advanced by the *médecins-dentistes* we find some truthful assertions,—viz.: That in order to practise dentistry upon a scientific basis it is necessary that the practitioner should possess a rather extensive medical education. As dentists are called upon to treat living organs, it is indispensable that they should thoroughly understand all the disturbances that may affect these organs, all the organic or extra-organic causes which may bring them about, and all the possible relations of dental and peridental diseases to the rest of the organism. If this be admitted, it follows as a rational consequence that the medical education of dentists must not be too limited nor too superficial, as in order to be in a position to understand thoroughly everything concerning dental diseases and their relations to morbid conditions of the organism it is necessary to possess a large share of medical science. I am therefore convinced that dental schools will have to extend gradually the scope of medical teaching, as the extent of the medical branches taught in dental schools at present is certainly insufficient. As an example, I will quote from the report of Dr. Charles Godon, of Paris, in his work, "Evolution de l'Art Dentaire."

From this report we gather that in the École Dentaire of Paris about four-fifths of the total time is devoted to technical teaching and only one-fifth to the scientific and medical subjects, to the importance of which we are now referring. If we consider that the dental course covers three years,—that is, about thirty-six months,—it is easy to see that by this arrangement the entire medical education required from the dental student could be given him in seven months, including in this time vacations and holidays. Now, you know that medical students who devote from five to six years to the study of this science leave the school after this time with a very incomplete knowledge of medicine. The time devoted to the teaching of medical branches in dental schools is very limited, and therefore the student can acquire only a very limited and superficial knowledge, which will not help him to understand the pathology of the dental and peridental organs and the complex relations which exist between these parts and the rest of the organism from the points of view of the etiology, pathology, and therapeutics of the disorders of which these organs may be the seat.

It is true that it has been decided to increase the course to four years, but, as far as I am aware, this fourth year will be devoted entirely to technical teaching; therefore even with a four years' course the medical education of dentists will remain just as imperfect and insufficient as it is at present. I believe in the necessity of extending the course to a five years' one, and during this period the dental student should be taught both general medicine and dentistry. I believe, further, that the curriculum should be arranged in such a way that the student might be able to devote at least four hours a day to medical subjects and the remaining five or six to strictly technical subjects. Five or six hours a day during five years should be sufficient to make skilful dentists, and, on the other hand, four hours a day devoted to medical subjects during five years should create dentists possessing a reasonable amount of medical education. They would have an amount of medical knowledge at least sufficient for the practice of their profession upon a scientific basis.

Regarding the question as to whether medical subjects should be taught in the dental schools or in the medical schools, I will positively answer that the medical subjects to be taught to dentists should be given by teachers making a specialty of these subjects in their bearing on dentistry. The necessity that the medical education of dentists should be a special one is certainly evident. Let us take, as an example, anatomy. On the bucco-facial region the anatomical training of the dentist should be more complete than that of the physician, just as, on the other hand, the anatomy of other regions could be suppressed from the curriculum or given in a more superficial way without at all injuring the dentist's education. For similar reasons, the teaching of other branches of the medical programme in dental schools should differ from the teaching of the same branches in the medical school.

To summarize, we will say that for dental students the course should be altogether a special one, and its purpose should be to impart to the student without the slightest loss of time the greatest amount of medical knowledge that will be useful to him in the practice of his profession.

I also wish to call your attention to the question of *preliminary educa-*

*tion.* Unfortunately, this question cannot be treated in a general way, because of the great differences presented by the organization of primary or elementary education, especially of the secondary education, in different countries; notwithstanding this difference in organization, however, I believe that the total duration of the studies that dental students should take up before entering institutions of higher education should not vary a great deal in the different countries. This involves, say, about twelve years. I believe that the preliminary education of dental students should be the same as that required from the physician, the lawyer, the engineer, etc. With a preliminary education of this sort lasting twelve years it is possible to undertake the study of dentistry after having acquired a good literary and scientific education, comprising among other things the study of ancient and modern history, physics, chemistry, natural history, mathematics, languages, etc. All these studies are apt to develop the intelligence of the student, and will serve the purpose of excellent preparation for the study of dentistry, permitting the dental student to assimilate easily the knowledge which will be imparted to him in the professional schools. The better educated the dentist is, the more will he be respected. It is essential that he should cease to be inferior to the physician from lack of either general culture or scientific requirements. He should rise to the same level as the physician, and should be prepared to intelligently discuss with him any scientific subject. He should be able to write correctly and easily, in order to be in a position to increase the literature of the profession by contributing works on dental topics. It would be a shame for the profession if the greatest part of its literature were written by physicians and not by dentists, as has already been asserted.

For the foregoing reasons I am led to express the wish that dental candidates should have a more complete preliminary education, and that in the curricula of dental schools more time should be devoted to the study of medical subjects.

Dr. Förberg then read the following report:

### DENTAL LEGISLATION IN SWEDEN.

The laws regulating dental practice in Sweden are very old, and as they have been considerably changed since the establishment of a dental institute, I deem it best to give only such points as will serve to make the subject clear. Since the foundation of this institute all dental instruction is given there, and the studies are obligatory.

Every one wishing to matriculate at this dental institute must present himself before the dean and produce his certificate of birth, testimonials as to his moral character, etc., from his clergyman, and a certificate from a high school that he has passed his final examination, which qualifies him to enter a university (maturity examination). This certificate must show that he obtained good marks in mathematics and physics, otherwise he must pass special examinations on these subjects.

There is only one dental institution in Sweden. It is the Dental Department of the Caroline Medico-Chirurgical Institute of Stockholm. It is a State institution. The course comprises three years, of about eight months, each year divided into two terms. During the first year the student attends the lectures in the medical department on medico-scientific subjects, in courses specially designed for dental students. At the end of the year the student is examined in these subjects. This is called the "dental candidate examination," and if passed entitles him to matriculation in the Dental Institute. Here he receives practical dental instruction for two years.

The final examination before becoming a legal dentist is given by the professors of the dental department and the professor in general surgery of the medical faculty as examiners, before an examining board consisting of the inspector (dean) and two practising dentists (chosen yearly) as censors. When the candidate has passed his examinations at the dental department he receives a certificate, and is referred to the Royal Medical Board, from which he receives his diploma as authorized dentist (tandläkare). The Royal Medical Board (Kongliga Medicinal Styrelsen) is therefore the licensing board.

Women have the same rights and privileges as men in regard to the study and practice of dentistry. Physicians have the right to practise dentistry without being obliged to pass any examination.

Dentists are allowed to prescribe drugs, and even poisons, for external use, but the administration of a general anæsthetic must take place in the presence of a physician.

There are about three hundred dentists in Sweden, of whom more than one-third are located in Stockholm. There are three Swedish dental journals,—*Nordisk Tandläkare-Tidskrift* and *Odontologisk Tidskrift,* of Stockholm; and *Reflector,* of Gothenburg. The Swedish dental societies are Svenska Tandläkare-Sällskapet, Goteborgs Tandläkare-Sällskapet, Skaraborgs Tandlakäre-Sällskapet, Malmö Tandläkare-Sällskapet, and Odontologiska Sällskapet. The members of the last four societies are at the same time members of the Svenska Tandläkare-Sällskapet and of the Scandinavian Dental Association, which is common for the three countries.

Dr. Jos. Arkövy presented a report embodying his views on the subject of dental education with especial reference to the three questions proposed for discussion by the International Commission of Education. This report is here omitted, as his views are fully expressed in his report published in the Proceedings of the International Dental Federation and International Commission of Education for 1901.

The following is the report submitted to the International Commission of Education by the Canadian representative, Dr. Charles E. Pearson:

REPORT ON THE THREE QUESTIONS PROPOSED BY THE INTERNATIONAL COMMISSION OF EDUCATION OF THE INTERNATIONAL DENTAL FEDERATION.

Regarding the three questions proposed by the International Commission of Education, your representative considers it advisable to quote from a very able article which appeared in the *Journal of the British Dental Association* for April and May, 1901. The writer of the article, A. E. Webster, D.D.S., M.D., professor of orthodontia and demonstrator in operative dentistry in the Royal College of Dental Surgeons, is editor of the *Dominion Dental Journal* and is a recognized authority on educational matters. The article referred to is detailed and complete, giving the full requirements, subject by subject, for matriculation to the school of the Royal College of Dental Surgeons, with examination papers, and a detailed statement of the course of studies in dentistry, also giving examination papers.

The quotations from this paper of Professor Webster's furnish answers to the three questions asked by the International Commission of Education.

*Question 1:* " What are the preliminary studies to be required of students before beginning their professional education?"

" The by-laws of the board of directors provide that every person who wishes to practise dentistry in Ontario shall matriculate in the Royal College of Dental Surgeons of Ontario. The board does not hold a matriculation examination, but accepts an official certificate of matriculation in arts in any Canadian or other recognized university, or of having passed the Ontario Educational Departmental junior or senior high school leaving examinations. These examinations are conducted by the Education Department, and are held simultaneously at every high school in the province once each year. All candidates write on the same examination papers. The answers are read and graded in the city of Toronto by teachers who gather there for that purpose. The junior leaving examination, which is the lowest standard of matriculation that the Royal College of Dental Surgeons will accept, embraces the following subjects and standards:

" *Part I., junior leaving standing.*—The subjects prescribed are reading, drawing, geography, botany (or agriculture), writing, with book-keeping and commercial transactions, English grammar, English literature, arithmetic and mensuration, English composition and history. *Part II.*—The subjects prescribed are English grammar and rhetoric, English composition, English literature, ancient history, arithmetic and mensuration, algebra, geometry, physics, and Latin, and one of the following groups: (*a*) French and Greek; (*b*) German and Greek; (*c*) French, German, and chemistry; (*d*) French, physics, and chemistry; (*e*) German and chemistry; (*f*) Botany, physics, and chemistry.

" The arts matriculation of the University of Toronto is based upon and is similar to that of the University of London, England."

*Question 2:* " Of what are dental studies to consist, how long ought they to last, and what should be the order of the programme?"

The course has been three terms of seven months' duration, but after

1902 it will be a four years' course of the same duration. The three years' course embraces the following:

First year, or freshman year: Anatomy (general). Fifty lectures. Minute anatomy of the bones and muscles of the head and neck.

Chemistry. Sixty lectures, including chemical physics, chemical nomenclature, symbols, combining weights and quantivalence and physical properties of the principal elements, and the preparation and chemical properties of oxygen, hydrogen, nitrogen, chlorine, sulphur, and potassium.

Histology. Thirty lectures, including general histology and minute histology of the human teeth.

Bacteriology. Twenty lectures and demonstrations.

Comparative dental anatomy. Ten lectures.

Operative technique. Fifteen lectures, embracing topographical anatomy of the human teeth; dental caries; discussion of filling-materials; cavity classification, root-canal medication and filling. Students must attend the technique laboratory eight hours each week during the session. In this course the student is expected to study the structure of the teeth and the anatomy of the surrounding parts. He must carve at least sixteen teeth from celluloid, making minute drawings of both longitudinal and cross sections of the teeth; practise root filling and medication; study and practise the use of instruments; form cavities in the carved celluloid teeth and fill them.

Prosthetic technique. Fifteen lectures, embracing the extraction of teeth, impressions, base-plates for artificial dentures, air-chambers, clasps, casts and dies, articulation of full dentures, manufacture of artificial teeth-crowns. Students must attend the prosthetic laboratory two hours each day of the session, and construct the following pieces: Partial upper denture in vulcanite; partial lower, with vulcanite clasps; partial upper, with metal clasps; repair vulcanite case; full upper and lower plain teeth, upper vulcanite, pink gum, air-chamber, lower cast metal; full upper and lower gum teeth, upper swaged aluminum, black vulcanite attachment, lower cast-metal base with pink gum; partial lower vulcanite with swaged metal stringer; partial upper swaged metal base; four gum teeth with rim and clasps, swaged upper metal base, wire edge, wire loops, air-chamber; Richmond crown; all-metal bicuspid crown; all-metal molar crown; metal bicuspid crown with porcelain face; Logan crown ground and set on a suitable root. Each case must be made to fit suitable models provided in the technique laboratory, and have a ticket attached to it when handed in for examination, to show that the demonstrator in charge has directed and seen the work in progress.

Metallurgy. Fifteen lectures, including the properties of metals, alloys, amalgams, lead, antimony, tin, bismuth, zinc, cadmium, copper, iron, aluminum, mercury, silver, iridium, palladium, platinum, gold.

Second year, or junior year: Operative dentistry. Fifty lectures on development of the teeth, clinical history and pathology of caries, pulpitis, pericementitis, and alveolar abscess; the composition and preparation of materials for filling teeth. The insertion of at least forty fillings in the teeth of patients in the infirmary.

Prosthetic dentistry. Fifty lectures. Extraction of teeth; composition and preparation of materials for taking impressions and for bases for artificial dentures; preparation of casts and dies for metal work; composition and manufacture of artificial teeth; the mechanical and æsthetic construction of dentures on plastic bases; the construction and insertion of at least five artificial dentures for patients in the infirmary.

Crown- and bridge-work. Sixteen lectures. The construction of two crowns and three bridges of three teeth each, on models prescribed by the professor in the department.

Orthodontia. Twelve lectures. A study of the materials used and their application; the construction and adaptation of one or more appliances to a suitable model.

Porcelain work. Five lectures.

Anatomy. Fifty lectures. Minute anatomy of the head and neck and general anatomy. Dissecting the head and neck, and at least one other part, at the medical department of the Provincial University.

Chemistry. Fifty lectures. The whole subject. Attendance in the chemical laboratory four hours a week throughout the session.

Physiology. Fifty-five lectures. Circulation, respiration, and digestion.

Medicine and surgery. Fifty lectures. Inflammation, healing and reparative processes, treatment of wounds, luxation and dislocation of the inferior maxilla.

Materia medica. Fifteen lectures. Weights and measures, source of preparation and mode of administration of, and medical and dental use of, one hundred drugs. Prescription-writing.

Third year, or senior year: Operative dentistry. Fifty lectures, embracing the whole subject and including dental pathology. Each student must insert for patients in the infirmary at least forty fillings, twenty-five of which must be gold; successfully devitalize and remove the pulps of five teeth and fill the root-canals; insert porcelain fillings as directed by demonstrators.

Prosthetic dentistry. Fifty lectures, covering the whole subject. Each student must construct and insert for patients in the infirmary at least five whole or partial dentures, and also make a gum-section denture on a prescribed model.

Crown- and bridge-work. The student must construct and insert at least three crowns, or a bridge of not less than three teeth for patients in the infirmary.

Orthodontia. Ten lectures, covering the whole subject. Each student must treat at least one case in the infirmary.

Dental therapeutics. Fifteen lectures, covering the whole subject.

Physiology. Fifty lectures, covering the general subject.

Medicine, surgery, and general pathology. Fifty lectures. Students must treat such cases as come to the infirmary, and attend once a week the clinics of the Victoria Hospital.

Dental metallurgy. Twenty-five hours in the laboratory.

Bacteriology. Twenty-five hours in the laboratory.

No candidate will be admitted to final examinations who does not present certificates to show that he has inserted for patients at least one hundred fillings and at least eight whole or partial dentures. Fifty of the fillings and six of the dentures may be inserted in the preceptor's office.

Candidates must obtain at least sixty per cent. of the marks allotted on all examinations in order to pass. All practical work done through the whole course is examined and graded, and is counted a part of the examination as much as the written papers.

Candidates of good moral character, twenty-one years of age, who have complied with the regulations and have satisfactorily passed all examinations, both written and practical, are awarded a certificate of license to practise dentistry in Ontario, and the title of L.D.S. (Licentiate of Dental Surgery).

*Question 3:* "What branches of the studies taught in medical schools must the student of dentistry follow?"

This question is answered best by studying what has been adopted by our school as stated in answer to Question 2.

### PROPOSED CURRICULUM.

In conclusion, a proposed curriculum for the four years' course of the Royal College of Dental Surgeons of Ontario is appended. This curriculum for the course of four sessions is proposed to come in force October 1, 1903:

*Add to present curriculum*—Physics, physical diagnosis, and anæsthesia; the practice of medicine, electro-therapeutics, institutes of dentistry to include history of dentistry, dental ethics, management of practice, advertising, etc.; laboratory, practical pathology; attendance at General Hospital.

*Re-arrange the work*—

First Year. Lectures: Histology, bacteriology, comparative dental anatomy, physics, materia medica, operative and prosthetic technics; commence anatomy. Laboratories: Histology, operative and prosthetic technics.

Second Year. Lectures: Therapeutics, orthodontia, crown- and bridge-work, complete anatomy, commence dental pathology, operative and prosthetic dentistry, commence chemistry. Laboratories: Practical anatomy, operative and prosthetic technics, crown- and bridge-work technic, orthodontia technic.

Third Year. Lectures: Metallurgy, electro-therapeutics, complete operative and prosthetic dentistry and dental pathology, complete chemistry, commence physiology, commence medicine and surgery, and general pathology. Laboratories: Infirmary, operative, prosthetic, orthodontia, crown- and bridge-work, chemistry, bacteriology, pathology, porcelain technic.

Fourth Year. Lectures: Complete physiology, complete medicine and surgery and general pathology, jurisprudence, physical diagnosis and anæsthesia, practice of medicine, institutes of dentistry. Laboratories: Infirmary, operative, prosthetic, orthodontia, crown- and bridge-work, porcelain work, chemical metallurgy, clinical medicine and surgery at the General Hospital.

### Dr. Rudolf Weiser presented the following report:

### REPORT ON THE THREE QUESTIONS PROPOSED BY THE INTERNATIONAL COMMISSION OF EDUCATION.

The first of the three questions proposed by the International Dental Federation reads as follows:

"(1) What are the preliminary studies to be required of students before beginning their professional education?"

There can be little doubt that all Austrian dentists are of the opinion that from one who intends to devote himself to dentistry the same preparatory education should be demanded as from one who will go to the university or to a polytechnic school, or who wishes to perform his military service as a one-year volunteer.

For my own part, I cannot but feel that the curriculum at most grammar schools and higher schools in most European countries loudly calls for thorough reform. In view of the increasing influence of sociology and the stagnation of parliamentarism, unceasing efforts are made to obtain for the members of the Sanitary Council such power in the departments of education as shall enable them to regulate the demands made upon the mental capacity of the young with due regard to the development of the rest of their organism, and to bring the ideals of mental education into harmony with the practical needs of political economy, instead of worshipping antiquated arrangements.

But the Austrian dentists are of the opinion that as long as admission to the university and the one-year voluntary service in the army is dependent on passing the matriculation examinations, the same should be required of those who intend to become dentists. For this opinion there are different reasons: First, the young and rising profession must desire that the social status of its representatives does not undeservedly sink below that of other branches of the art of healing. We dentists will not profane the spiritual inheritance of a Wedl, Salter, Heath, Garretson, Tomes, Leber, Rottenstein, Magitot, Mallasez, Muehlreiter, Albrecht, Mills, Underwood, Mummery, Miller, Zuckerkandl, Taft, Kingsley, Bonwill, Martin, Case, Essig, Brophy, Arkövy, Rothmann, Partsch, Sachs, Scheff, von Metnitz, Bödeker, Walkhoff, Wetzel, Ebner, Schaber, Zsigmondy, Gis, Roese Roemer, Preiswerk, and innumerable other researchers and promoters of our branch.

It certainly cannot be good for our science that less capable persons should receive official permission to enter it. Our calling cannot be raised in the opinion of the public, and of our colleagues the medical men, if in a country with compulsory military service the dentists should rank lower than the veterinary surgeons, and if practical dentistry is the refuge of such as do not get on well at the grammar school or the higher school.

Of not less importance is the moral factor, which is tellingly pointed out by the German teachers of dentistry in a memorandum to the Bundesrath of the German Empire. They say, "The demand for matriculation gains increased importance through the consideration that all the knowledge and accomplishments, the most thorough instruction, can be made useful only if the dentist, beginning practice with a really high conception of his calling and with a large measure of faithfulness to duty, undertakes his task of making the progress of modern dentistry useful to his patient,—undertakes it with the moral maturity which is generally to be expected of older persons. Under present circumstances young men mostly enter the first class of the grammar school in their sixteenth or seventeenth year. Then they begin their dental studies, and mostly conclude them after three years, when they are in their twentieth year. Even supposing they then become assistants for two or three years,—which is the case with only a fraction of them,—they become independent at a time when in general they do not yet possess the manly stability and the mature gravity which are demanded by intercourse with the public and by responsibility in difficult situations, *e.g.*, administering narcotics, and in the carrying out with proper insistence the demands of dental science. No wonder, then, that many a one stumbles, and to his own detriment gives way to dangerous temptations; too often he has to lament that in his practice he should have turned from the creditable principles which he brought with him from the university, and should have taken to greedy bleeding of the public because in that way it seemed possible to make money more quickly and easily. The measure of care, perseverance, thoroughness, and faithfulness to duty possessed by a conscientious dentist is particularly large; the great pains and the expenditure of time necessary—and which at the moment, considering their object, seem extravagant—often tempt the less serious, immature characters to neglect their duty, and all too often, instead of painstaking, thorough work, which alone can be successful, there is a scamping done with dazzling facility and rapidity."

Questions 2 and 3 of the International Commission of Education are—

"(2) Of what are dental studies to consist, how long ought they to last, and what should be the order of the programme?"

"(3) What branches of the studies taught in medical schools must the student of dentistry follow?"

For the Austrian the answering of these two questions becomes decidedly easier, and the report will seem clearer if the order of the questions be altered.

Since the Austrian dentists agree in the principle that dentistry forms a branch of the healing art in general, which demands its special education and training, the matter to be presented now resolves itself into the discussion of Question 3.

As was referred to in my report in collaboration with Dr. Zsigmondy to the International Dental Federation, London, 1901, I here again give expression to the conviction that the unexpected and enormous development of the entire healing art, as a branch of human knowledge which it is quite impossible for one person to master completely, loudly calls for a reorganization of the course of medical study. On the part of students and practitioners it is sought, by the requirements of the people one is compelled, to give the medical student first of all a three years' fundamental education, and then to divide the remainder—say two years—of his course into branch subjects.

A common medical foundation for all those who later will devote themselves to a special branch of medicine affords, first of all, the advantage of admitting into the ranks of the profession only educated doctors with a wide mental horizon, and those whose powers of observation have been guided into the right channels; and secondly, such a foundation enables the student to decide on this special branch only after a deeper insight into the situation, and enables him with insignificant loss of time to change, if at the beginning of his special studies he should find that he has not chosen in accordance with his own inclinations and abilities.

In my opinion this general training to be demanded of all physicians would have to comprise the following departments: Descriptive anatomy, connected with practice in dissecting, physiology, histology, bacteriology, pathological anatomy, general experimental pathology, internal medicine, pharmacology, surgery, skin diseases, and syphilis.

It would be exceeding the limits of this report were I to point out in detail how important is each of the subjects above mentioned to him who wishes to have a real right to the name of doctor. The only argument that I will bring forward for the importance of the demand is that he who inflicts wounds for curative reasons should be acquainted with the course of the arteries, with the lymphatic glands, with the stopping of bleeding, the nature of wounds, and their treatment. The proposal that one and the same patient be treated by two or more specialists would be practicable sometimes in large towns, but not in small ones, and the simultaneous treatment by several doctors will meet with pecuniary difficulties as well as those of locality and time.

Having regard to our responsibility to our patients and the judicial authorities, the demand that medical practitioners should be acquainted with the appearance of syphilis and with the measures for protecting against its transmission is certainly amply justified.

As to the question, " Of what are dental studies to consist, how long ought they to last, and what should be the order of the programme?" On the ground of my own experience that even very talented and diligent Austrian physicians who, after finishing their medical studies, have devoted themselves to dentistry cannot without danger be employed in a good practice in a large town before the expiration of two or three years, I think it necessary that students' theoretical and practical instruction in the purely dental subjects (that is, after completing fundamental studies, lasting four or six terms) should comprise five or six terms.

First Year. Morning: Descriptive anatomy, with practice in dissection; physiology; bacteriology. Afternoon: Practice in chemical laboratory; histology and microscopy.

Second Year. Morning: General and experimental pathology; pathological anatomy; treatment of internal disorders. Afternoon: Pharmacology; afternoon visit to the clinic for internal diseases; theoretic lectures on dental surgery.

Third Year. Morning: General surgery; special surgery of the mouth and maxillary sinus; skin diseases and syphilis; practical exercises in the preservative treatment of the teeth; phantom. Afternoon: Practical exercise in the surgery of the mouth and maxillary sinus (anæsthesia); practice in topographo-anatomical dissection (throat, mouth, and jaw); practical exercise in the surgery of the mouth and maxillary sinus (narcosis).

Fourth Year. Morning (8 to 9): Dental metallurgy; (9 to 12) practice in conservative dentistry (on the patient). Afternoon (1 to 3): Practice in the surgery of the maxillary sinuses (anæsthesia); (3 to 7) practical exercise with artificial teeth and jaws.

Fifth Year. Morning (8 to 11): Practical exercise in conservative dentistry; (11 to 1) orthodontia. Afternoon: Crown- and bridge-work; obturators; mandibular splints. Examinations and services as demonstrators for the students of lower years.

A glance at this curriculum shows—

(1) That a dentist who has passed through it will be thoroughly competent to deal with every case that comes within the limits of his profession; it allows—

(2) Of the student's altering his plan of study in his third year without incurring thereby any serious loss of time or material disadvantage, if he should at that stage of his career have come to the conclusion that he has no vocation for the profession of dentistry; it also makes possible—

(3) The completion of the course of study in eight terms, if the educational authorities should think fit to sanction such an education on the ground of pecuniary difficulties, in exceptional cases.

This curriculum would have for its object the giving of exhaustive and detailed instruction, both theoretical and practical, in the following branches of technical knowledge: Theoretical lectures on the means known to dental surgery for the preservation of the teeth, and including the art of preparing and fitting artificial teeth and jaws; special surgery of the mouth and maxillary sinus, including practical exercises; practical exercises in the preservative treatment of the teeth; practical instruction in the preparation and fitting of artificial teeth and jaws; orthodontia; dental metallurgy; electrology.

The author of this report has ventured to conclude it by laying before his readers an approximate suggestion for a curriculum drawn up as a test for himself, to show whether his demands, as explained and elaborated above, could be brought into harmony with actually existing circumstances.

(To be continued.)

